# Novel surgical approaches for treating myopic traction maculopathy: a meta-analysis

**DOI:** 10.1186/s12886-024-03374-0

**Published:** 2024-03-05

**Authors:** Miguel A. Quiroz-Reyes, Erick A. Quiroz-Gonzalez, Miguel A. Quiroz-Gonzalez, Virgilio Lima-Gomez

**Affiliations:** 1 Oftalmologia Integral ABC, Retina Department, Medical and Surgical Assistance Institution (Nonprofit Organization) affiliated with the Postgraduate Studies Division at the National Autonomous University of Mexico, Lomas de Chapultepec, Lomas de Chapultepec, Mexico City, 11000 Mexico; 2 Institute of Ophthalmology, Chimalpopoca 14, 06800 Mexico City, Colonia Obrera Mexico; 3 Juarez Hospital, Colonia Magdalena de Las Salinas, Av. Politecnico Nacional 5160, 07760 Mexico City, Mexico

**Keywords:** Fovea-sparing internal limiting membrane peeling, Foveal retinal detachment, Myopic foveoschisis, Full-thickness macular hole, High myopia, Myopic traction maculopathy, Outer lamellar macular hole, Macular retinoschisis, Pathologic myopia, Posterior staphyloma

## Abstract

**Background:**

Myopic traction maculopathy (MTM) is a complication of pathological myopia and encompasses various pathological conditions caused by tractional changes in the eye. These changes include retinoschisis, foveal retinal detachment, and lamellar or full-thickness macular holes (FTMHs). This meta-analysis evaluated the safety and efficacy of novel surgical for treating MTM.

**Methods:**

To compare the outcomes of different surgical approaches for MTM, multiple databases, including Web of Science, PubMed, Scopus, ClinicalTrials.gov, the Cochrane Central Register of Controlled Trials, Ovid MEDLINE, Embase, and the Meta-Register of Controlled Trials, were comprehensively searched. The meta-analysis was performed using RevMan 5.1.

**Results:**

Nine comparative studies involving 350 eyes were included in this meta-analysis. There were significant differences between fovea-sparing internal limiting membrane peeling (FSIP) and standard internal limiting membrane peeling (ILMP). Preoperative best-corrected visual acuity BCVA (standard mean difference (SMD): -0.10, 95% CI: -0.32 to 0.12) and central foveal thickness CFT (SMD: 0.05, 95% CI: -0.22 to 0.33) were not significantly different (*p* = 0.39 and *p* = 0.71, respectively). However, the postoperative BCVA improved significantly (SMD = − 0.47, 95% CI: − 0.80, − 0.14, *p* = 0.006) in the FSIP group compared to the standard ILMP group. Postoperative CFT did not differ significantly between the two groups (*p* = 0.62). The FSIP group had a greater anatomical success rate than the other groups, although the difference was not statistically significant (*p* = 0.26). The incidence of postoperative macular hole formation was significantly lower (OR = 0.19, 95% CI = 0.07–0.54; *p* = 0.05) in the FSIP group than in the standard ILMP group. The unique characteristics of highly myopic eyes, such as increased axial length and structural changes, may have contributed to the greater incidence of FTMH in the ILMP group.

**Conclusion:**

Based on the findings of this meta-analysis, FSIP is the initial surgical approach for early-stage MTM and has shown promising outcomes. However, to establish the safest and most efficient surgical technique for treating different MTM stages, further comparative studies, specifically those focusing on ILMP and FSIP, are necessary.

**Trial registration:**

Retrospectively registered.

**Supplementary Information:**

The online version contains supplementary material available at 10.1186/s12886-024-03374-0.

## Background

Myopic traction maculopathy (MTM) is characterized by significant retinoschisis-like thickening of the outer retina accompanied by posterior staphyloma (PS) in highly myopic eyes [1]. MTM is considered a major complication of pathologic myopia (PM) and is projected to become a leading cause of visual impairment worldwide in the coming decades [2, 3]. The prevalence of visual impairment and blindness associated with PM ranges from 12 to 27% in Asian populations and 7% in Western populations [3–6]. MTM has been identified and categorized via the extensive application of OCT and vitrectomy techniques [7]. Additionally, studies have reported the occurrence of epiretinal membranes (ERMs), macular retinoschisis (RS), stretched retinal vessels, different degrees of posterior staphyloma (PS), outer lamellar macular holes (MHs), full-thickness MHs (FTMHs), abnormally rigid inner limiting membrane (ILM), foveal retinal detachment (FRD), and MH retinal detachment (MHRD) [3, 8–10]. MTM progression is driven by increasing traction exerted on these structures [11].

The ab externo surgical approach for MTM was introduced long before its definition in 1930. In terms of treatment, surgery is recommended for patients with reduced visual acuity, detached fovea, FTMH, or champagne-flute-shaped retinoschisis and MHRD [12, 13]. Surgical treatment options include pars plana vitrectomy (PPV) with or without ILM stripping and macular buckling (MB) [14, 15]. In 1957, Schepens et al. proposed the MB technique, which has since been considered the best surgical approach for the treatment of myopic MHRD [16]. In 2012, Shimada et al. presented an innovative approach to myopic FRD surgery known as fovea-sparing internal limiting membrane peeling (FSIP). In contrast to the standard ILM peeling (ILMP) technique, the FSIP technique retains a portion of the ILM attached to the fovea, demonstrating greater efficacy in preventing MH development [17, 18]. Recently, several novel surgical methods, including autologous neurosensory retinal-free patch transplantation [19], lens capsular flap transplantation [20], ILM repositioning with autologous blood clotting (ABC) [21] and inverted ILM insertion [22], have been proposed for MTM. According to Michalewska et al., the postoperative closure rate using the inverted ILM flap technique was better (98%) for large idiopathic MHs than for small idiopathic MHs [23]. Following the same procedure as that used for myopic MH treatment, Kuriyama et al. [22] reported a closure rate of 80%. Recently, Chen et al. [24] reported a closure rate of 100% using inverted ILM insertion for MHRD in highly myopic patients.

Numerous surgical techniques are available for the treatment of MTM, but there is no standard treatment approach. Recent advancements in various surgical techniques have introduced new viable options for MTM treatment [25]. However, it is necessary to thoroughly examine these studies to validate their effectiveness and ensure their methodological rigor in assessing the efficacy of surgical treatments for MTM. Careful evaluation of their methodology, study design, and outcome assessments is required to derive compelling and coherent conclusions regarding their impact on patients. Therefore, we conducted a comprehensive review and meta-analysis of clinical studies involving MTM patients who underwent surgical treatment. This study aimed to compare the outcomes of different surgical treatments for MTM. Numerous studies have reported that the application of the FSIP technique for treating MTM leads to improved visual and anatomical outcomes [26, 27]. This meta-analysis aimed to increase the sample size, obtain more reliable findings to validate previous studies, and provide updates on the preferred surgical approach based on recently published studies.

## Methods

### Search strategy

The search was conducted in accordance with the Preferred Reporting Items for Systematic Reviews and Meta-Analyses (PRISMA) guidelines. Database searches of the literature were conducted by using PubMed, ClinicalTrials.gov (www.clinicaltrials.gov), Scopus, Web of Science, and the Cochrane Central Register of Controlled Trials (CENTRAL), which contains the Cochrane Eyes and Vision Group Trials Register (*The Cochrane Library* 2013, Issue 2); Ovid MEDLINE; Ovid MEDLINE In‐Process and Other Non-Indexed Citations, Ovid MEDLINE Daily; Ovid OLDMEDLINE (January 1990 to October 2020); Embase (January 2000 to October 2022); and the *meta*Register of Controlled Trials (*m*RCT) (www.controlled-trials.com). In all of these databases, specific keywords were used to narrow the results to the desired literature (Supplementary file). The reference lists of the studies included in the review were also searched for information on other studies on the use of ILMPs for the surgical treatment of MTM. The databases were ultimately searched on October 12, 2022, and an additional search was performed using Google Scholar to identify the reference lists of the originally identified articles. No language restrictions were placed on the electronic searches for the trials.

### Screening of the retrieved studies

Two authors screened studies using the Covidence.org tool. The title and abstract of each publication were reviewed by two reviewers (MAQR and EAQG)*,* who selected the studies relevant to our desired objectives. Following the initial screening, disagreements were discussed and resolved by the two reviewers. If an agreement could not be reached, a third reviewer (VLG) was consulted to arbitrate the study.

### Study selection criteria

Studies involving individuals with highly myopic eyes, defined by an axial length (AL) longer than 26.5 mm or a refractive error of more than -6.0 diopters, who exhibited myopic foveoschisis leading to gradual vision loss were included in the study. The detailed inclusion and exclusion criteria were as follows:

**Table:** 

Criteria Type	Inclusion Criteria	Exclusion Criteria
Study Design	Comparative Prospective and retrospective studies, Conference Abstracts with all the required data	Studies such as noncomparative studies, single-arm studies, animal studies, conference abstracts with limited data or that present data from previous studies, review articles, doctoral dissertations, articles that present data from the same study, and case reports
Surgical Approach	Vitrectomy using various techniques, including the classical ILMP technique, FSIP techniques, inverted ILM insertion, ILM flap with ABC technique, lens capsule transplantation, multilayered inverted ILM (ML-IILM) techniques, autologous neurosensory retina grafting techniques, and human amnion membrane grafting (AMG) technique	Use of macular buckling techniques
Publication Language	English	Other than English
Follow-up durations	More than or equal to 6 months	Less than 6 months

### Types of intervention

The following intervention comparisons were considered: standard ILMP technique versus FSIP, inverted ILM insertion versus ILM flap with the ABC technique, lens capsule transplantation versus ML-IILM techniques, and autologous neurosensory retinal grafting (ARG) techniques versus the human AMG technique.

### Outcome measures

The primary outcomes of the study were as follows: (a) visual outcome measures, that is, postoperative changes in best-corrected visual acuity (BCVA); (b) anatomic outcome measures, that is, the proportion of patients with foveal (retinal) reattachment and the mean change in central foveal thickness (CFT); and (c) the postoperative incidence of MHs and complications.

### Data extraction

Each article was evaluated after a database search to determine whether it was unquestionably relevant, perhaps relevant, or certainly irrelevant. All the articles were checked for inclusion or exclusion after duplicates were removed. The study design, reports, and final results of all the included studies were thoroughly reviewed. Two surgeons (MAQR and EAQG) checked all the publications retrieved during the search, selected studies that met the inclusion criteria, and gathered data from those studies. The name of the first author, year of publication, number of participants in each group, refractive error, axial length, preoperative and postoperative BCVA, percentage of patients with CR and MH, mean change in CFT, and length of follow-up were extracted from the list of items.

### Methodological assessment and statistical analysis

We used a modified checklist derived from the Newcastle–Ottawa Scale (NOS) to assess the quality of the studies included in the meta-analysis. The assessment encompassed three categories: selection, comparability, and exposure/outcome. Each category comprised specific criteria, with studies scoring points based on adherence to these criteria [20]. A nine-point scale was used for evaluation, with studies categorized as high, medium, or poor quality based on their scores. Studies meeting a threshold of > 4 points on the NOS were considered for the final analysis, and those scoring < 3 points were excluded. Additionally, the quality of the randomized controlled trials (RCTs) was evaluated using the 5-point Jadad scale.

### Statistical analysis

To assess heterogeneity among studies, Cochrane's Q statistic and the I^2 statistic were used. The Cochrane Review Manager (RevMan) software was used to analyze continuous variables via weighted mean difference (WMD) calculations and computed odds ratios (ORs) for dichotomous variables. Confidence intervals (CIs) were calculated using an established methodology [28]. Publication bias was examined using funnel plots and tested via Begg’s rank correlation and Egger’s linear regression tests, with significance set at *p* < 0.05 [29, 30].

## Results

**Fig. 1:**
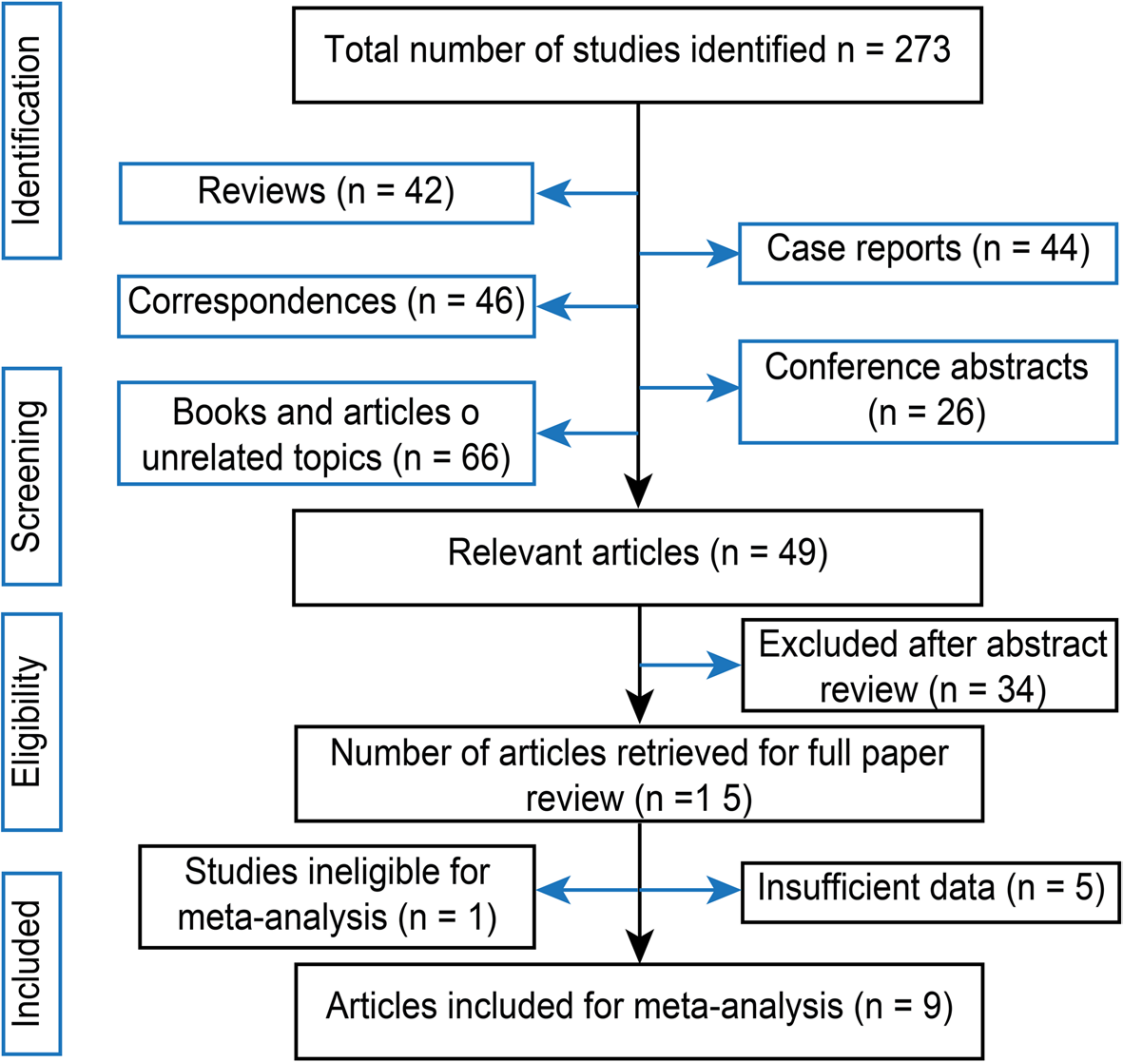
PRISMA flow diagram of all the retrieved articles that were included in this meta-analysis

After the database searches, 273 articles were retrieved using various keywords. Initially, reviews, case reports, correspondences, abstracts, and irrelevant documents were excluded. Subsequently, 34 additional studies were excluded after screening the titles and abstracts. Among the remaining studies, five were excluded because of insufficient data and irrelevant interventions. Upon assessment of the full text, 10 English studies were deemed eligible for meta-analysis (Fig. 1). Of these, nine studies compared standard ILMP to FSIP, while one study compared inverted ILM insertion versus the ILM flap with the ABC technique; these studies were not included in the meta-analysis. All nine selected studies were comparative nonrandomized and retrospective studies. No other novel comparative interventions were identified in this study.

### Characteristics of the included studies

**Fig. 2:**
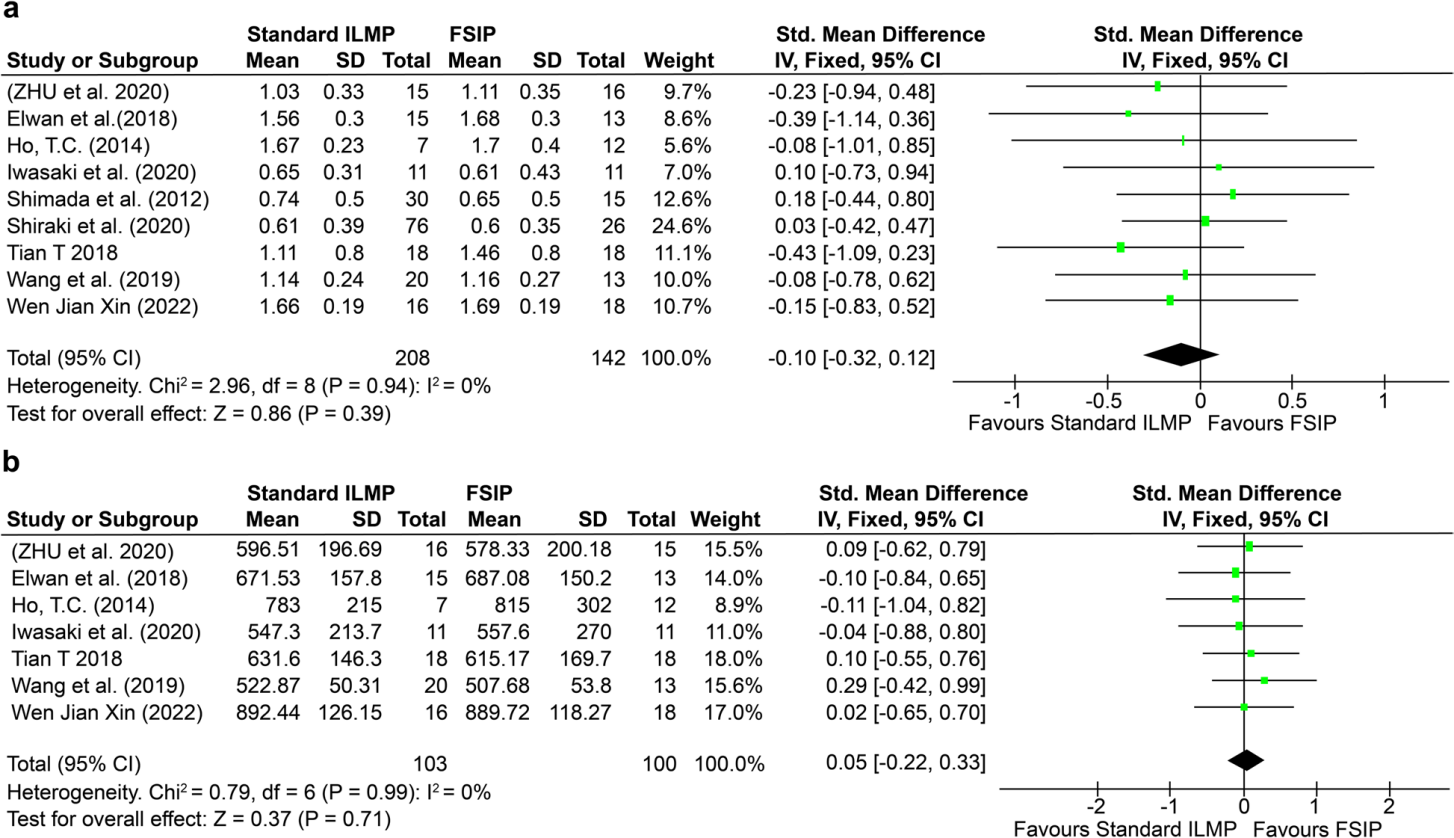
**a** Forest plot showing the preoperative BCVA in MTM patients in the FSIP group and the standard ILM peeling group; **b** Forest plot showing the preoperative central foveal thickness (CFT) in MTM patients in the FSIP group and the standard ILM peeling group

A total of 350 eyes were included in the nine studies. Approximately 141 eyes underwent FSIP, and 209 eyes underwent standard ILMP. Eight studies were retrospective, whereas one study was prospective. The detailed characteristics of the studies are presented in Table 1. The results of the quality assessment are presented in Table 2. All the included patients were diagnosed with MTM and had an AL > 26.5 mm. Moreover, there were no significant differences in the preoperative BCVA or CFT between the two groups, as shown in Fig. 2(a) and (b). (BCVA: standardized mean deviation (SMD): -0.10, 95% CI = -0.32 to 0.12, *p* = 0.39; CFT: SMD: 0.05, 95% CI = -0.22 to 0.33, *p* = 0.71).

**Table 1:** Characteristics of the studies included in the meta-analysis

Study design	Study conducted by	No of eyes	Average age in years	AL (mm)	Treatment	ILM staining	Tamponade	Preoperative foveal retinal detachment (%)	Preoperative ERM	Preoperative- CFT (μm)	Postoperative- CFT (μm)	Preoperative BCVA (LogMAR)	Postoperative BCVA (LogMAR)	Preoperative MH	Postoperative MH	Follow-up (months)
Retrospective	Xin et al. [31]	18	56.78 ± 5.75	27.59 ± 0.57	FSIP	Indocyanine green	Air	5 (27.78)	-	889.72 ± 118.27	134.94 ± 21.49	1.69 ± 0.19	1.13 ± 0.21	0	0	6 months
		16	57.75 ± 6.37	27.55 ± 0.70	Standard ILMP	Indocyanine green	Air	3 (18.75)	-	892.44 ± 126.15	149.44 ± 19.60	1.66 ± 0.19	1.45 ± 0.11	0	2	6 months
Retrospective	Wang et al. [13]	15	65.8 ± 7.1	29.1 ± 1.1	FISP	Indocyanine green	Gas	15	-	-	-	0.65 ± 0.5	0.34 ± 0.4	0	0	11.3 ± 5.3
		30	63.2 ± 12.0	29.1 ± 1.8	Standard ILMP	Indocyanine green	Gas	30	-	-	-	0.74 ± 0.5	0.58 ± 0.6	0	5	47.5 ± 18.5
Prospective	Elwan et al. [32]	13	54.54 ± 6.4	29.2 ± 9.05	FSIP	Brilliant blue G (BBG)	Gas	-	-	687.08 ± 150.20	168.38 ± 44.41	1.68 ± 0.3	0.7 ± 0.26	0	0	12 months
		15	54.67 ± 5.7	29.15 ± 11.87	Standard ILMP	Brilliant blue G (BBG)	Gas	-	-	671.53 ± 157.8	156.47 ± 45.8	1.56 ± 0.3	0.83 ± 0.23	0	0	12 months
Retrospective	Iwasaki et al. [33]	11	71.8 ± 6.7	29.7 ± 1.8	FSIP	Brilliant blue G (BBG)	Gas	6 (54.5)	8 (72.7)	557.60 ± 270.00	128.80 ± 46.50	0.61 ± 0.43	0.34 ± 0.42	0	0	17.1 months
		11	70.4 ± 9.7	28.8 ± 1.6	Standard ILMP	Brilliant blue G (BBG)	Gas	5 (45.5)	6 (54.5)	547.30 ± 213.70	130.30 ± 38.00	0.65 ± 0.31	0.52 ± 0.43	0	3	40.3 months
Retrospective	Wang et al. [34]	13	50.0 ± 9.6	29.3	FSIP	Brilliant blue G (BBG)	C3F8 gas	6 (46)	0	507.68 ± 53.80	308.30 ± 65.45	1.1 (0.8–1.7)	0.9 (0.6–1.2)	0	1	6 months
		20	50.1 ± 9.3	28.95	Standard ILMP	Brilliant blue G (BBG)	C3F8 gas	8 (40)	0	522.87 ± 50.31	312.94 ± 45.50	1.1 (0.8–1.7)	0.9 (0.7–1.2)	0	2	6 months
Retrospective	Shiraki et al. [35]	26	65.2 ± 10.8	30.1 ± 1.9	FSIP	Brilliant blue G (BBG)	Sulfur hexafluoride gas	8	-	-	-	0.60 ± 0.35	0.32 ± 0.43	12	0	21.5 ± 9.0
		76	65.5 ± 9.3	29.3 ± 1.4	Standard ILMP	Brilliant blue G (BBG)	Sulfur hexafluoride gas	29	-	-	-	0.61 ± 0.39	0.37 ± 0.38	0	6	25.8 ± 10.3
Retrospective	Tian et al. [27]	18	52.80 ± 10.80	-	FSIP	Brilliant blue G (BBG)	Fluid air exchange	10 (55.6)	-	615.17 ± 169.7	143.36 ± 52.40	1.46 ± 0.80	0.56 ± 0.30	0	3	20.9 ± 9.6
		18	58.00 ± 13.20	-	Standard ILMP	Brilliant blue G (BBG)	Fluid air exchange	9 (50)	-	631.60 ± 146.30	141.60 ± 93.40	1.11 ± 0.80	0.67 ± 0.50	1	1	19.6 ± 4.1
Retrospective	Ho et al. [26]	12	58.20 ± 10.50	-	FSIP	Indocyanine green	-	4	-	815.00 ± 302.00	122.00 ± 67.00	1.70 ± 0.40	0.89 ± 0.56	-	0	55.6 ± 16.2
		7	54.40 ± 6.50	-	Standard ILMP	Indocyanine green	-	7	-	783.00 ± 215.0	137.00 ± 52.0	1.67 ± 0.23	1.39 ± 0.33	-	2	52.4 ± 14.6
Retrospective	Zhu et al. [36]	15	57.55 ± 9.45	-	FSIP	-	-	-	-	578.33 ± 200.18	134.27 ± 25.29	1.11 ± 0.35	0.64 ± 0.21	-	0	18.65 ± 5.15 months
		16		-	Standard ILMP	-	-	-	-	596.51 ± 196.69	126.25 ± 36.61	1.03 ± 0.33	0.67 ± 0.24	-	1	

The numbers in the "postoperative macular hole (MH)" column represent the number of eyes with postoperative macular hole (MH) formation. Specifically, 0 corresponds to patients with no MH, 1 indicates one patient, and 2 indicates two patients with MH

*FSIP* Fovea-sparing internal limiting membrane peeling, *standard ILMP* standard internal limiting membrane peeling, *AL* Axial length, *ERM* Epiretinal membrane, *CFT* Central foveal thickness, *BCVA* Best corrected visual acuity

**Table 2:** MINORS for assessing the quality of included studies

**Methodological item for nonrandomized studies**	Elwan et al. [32]	Wang et al. [13]	Shiraki et al. [35]	Wang et al. [34]	Iwaski et al. [33]	Xin et al. [31]	Tian et al. [27]	Ho et al. [26]	Zhu et al. [36]
1. A clearly stated aim	2	2	2	2	2	2	2	2	2
2. Inclusion of consecutive patients	2	2	2	2	2	2	2	2	2
3. Prospective collection of data	2	0	0	0	0	0	0	0	0
4. Endpoints appropriate to the aim of the study	2	2	2	2	2	2	2	2	1
5. Unbiased assessment of the study endpoint	1	1	1	1	1	1	1	1	1
6. Follow-up period appropriate to the aim of the study	2	2	2	2	2	2	2	2	2
7. Loss to follow up less than 5%	2	2	2	2	2	2	2	2	2
8. Prospective calculation of the study size	0	0	0	0	0	0	0	0	0
9. An adequate control group	2	2	2	2	2	2	2	2	1
10. Contemporary groups	2	1	2	2	2	2	2	2	1
11. Baseline equivalence of groups	2	2	2	2	2	2	2	2	2
12. Adequate statistical analyses	2	2	1	2	2	2	2	2	1
13. MINORS score	20	18	18	19	19	19	19	19	15

### Visual outcome efficacy analysis

**Fig. 3:**
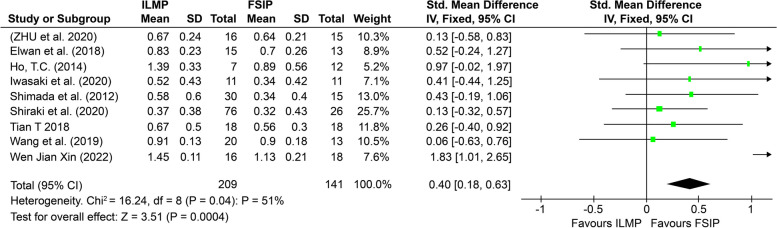
Forest plot showing the difference in postoperative BCVA between MTM patients in the FSIP group and those in the standard ILM peeling group

After analyzing all the included studies, we found that BCVA improved postoperatively in both groups, excluding patients who developed MHs. However, the overall improvement across both groups was not statistically significant. The postoperative BCVA showed a greater change in the FSIP group than in the standard ILMP group. As shown in Fig. 3 (SMD = − 0.47, 95% CI: − 0.80, − 0.14, *p* = 0.006), the postoperative increase in BCVA was highly significant.

### Measures of anatomic outcomes

**Fig. 4:**
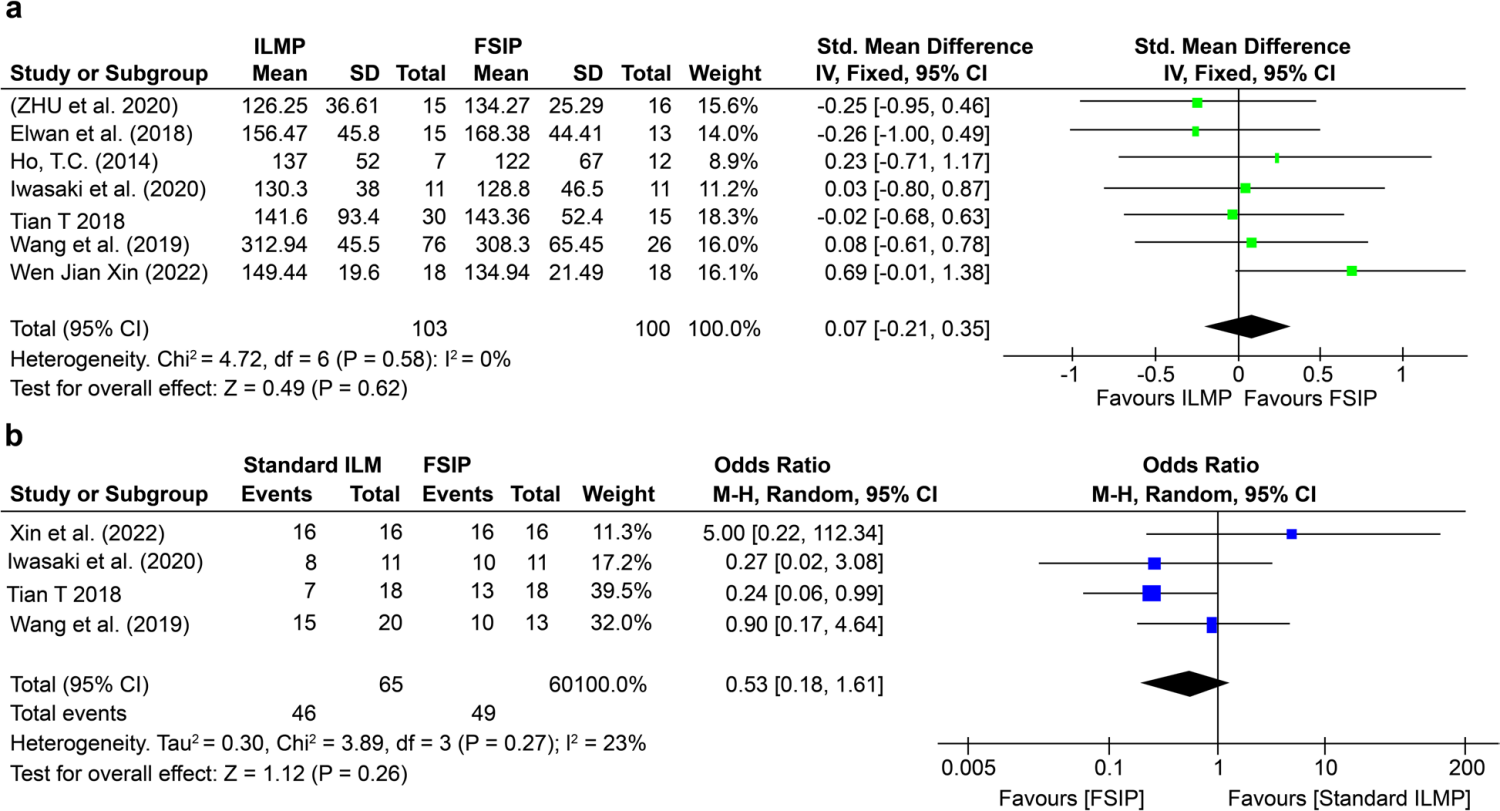
**a** Forest plot showing the difference in postoperative CFT between the FSIP group and the standard ILM peeling group of MTM patients. **b** Forest plot showing the difference in the rate of anatomic success between the FSIP group and the standard ILM peeling group

In six studies [27, 31–34, 36] involving 201 eyes, there was a significant decrease in postoperative CFT. However, in this meta-analysis, we did not find a statistically significant difference between the two groups, as shown in Fig. 4(a) (SMD = 0.07, 95% CI = -0.21 to 0.35; *p* = 0.62). Four studies [13, 26, 31, 34] reported the rate of anatomic success in 125 eyes and reported that the standard ILMP method was associated with a significantly greater probability of anatomic success (Fig. 4(b)) (OR = 0.53, 95% CI = 0.18 to 1.61; *p* = 0.26). These findings suggest that postoperative MH formation is greater after standard ILMP than after FSIP.

### Postoperative complications

**Fig. 5:**
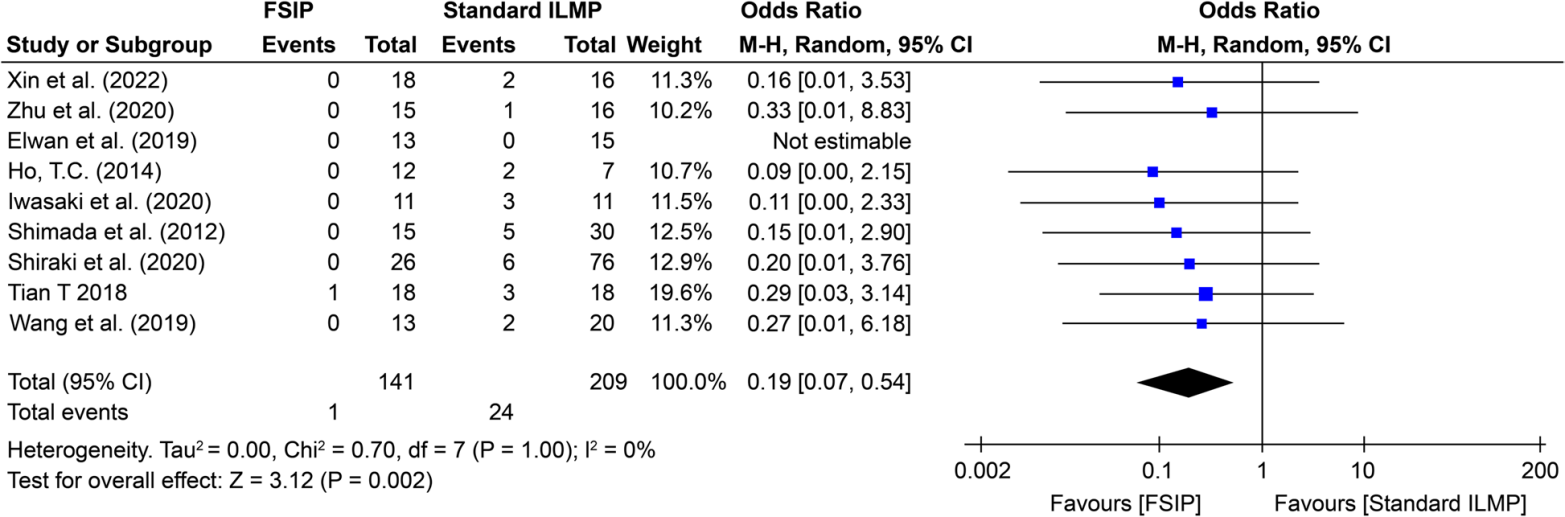
Forest plot showing the proportion of macular hole (MH) formation between the FSIP group and the standard ILM peeling group

Eight studies reported postoperative complications, such as MH and RD, in patients who underwent FSIP compared to those who underwent standard ILMP. The most serious postoperative complication was MH formation; no intraoperative complications were reported. After excluding two studies [32, 36], the remaining five studies [26, 27, 31, 33–35] showed that FSIP was significantly superior to standard ILMP for preventing MH formation (OR = 0.19, 95% CI = 0.07–0.54; *p* = 0.05), as shown in Fig. 5. One study reported no postoperative complications [13].

### Sensitivity analysis

**Fig. 6:**
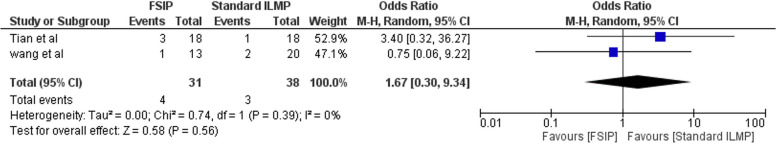
Forest plot showing the sensitivity analysis with a *p* value of 0.56

We conducted sensitivity analyses to examine the impact of FSIP on vision, specifically excluding individuals without postoperative MHs from the analysis. The results did not reveal any significant change in vision (*p* = 0.56), and the odds ratio was 1.67, with a 95% confidence interval of 0.30–9.34. The I^2^ statistic, which measures the percentage of variability across studies due to heterogeneity rather than chance, was 0%, indicating a low variability (Fig. 6). These findings suggest that even in patients without postoperative MH, the positive impact of FSIP on vision was not significantly different.

### Publication bias

**Fig. 7:**
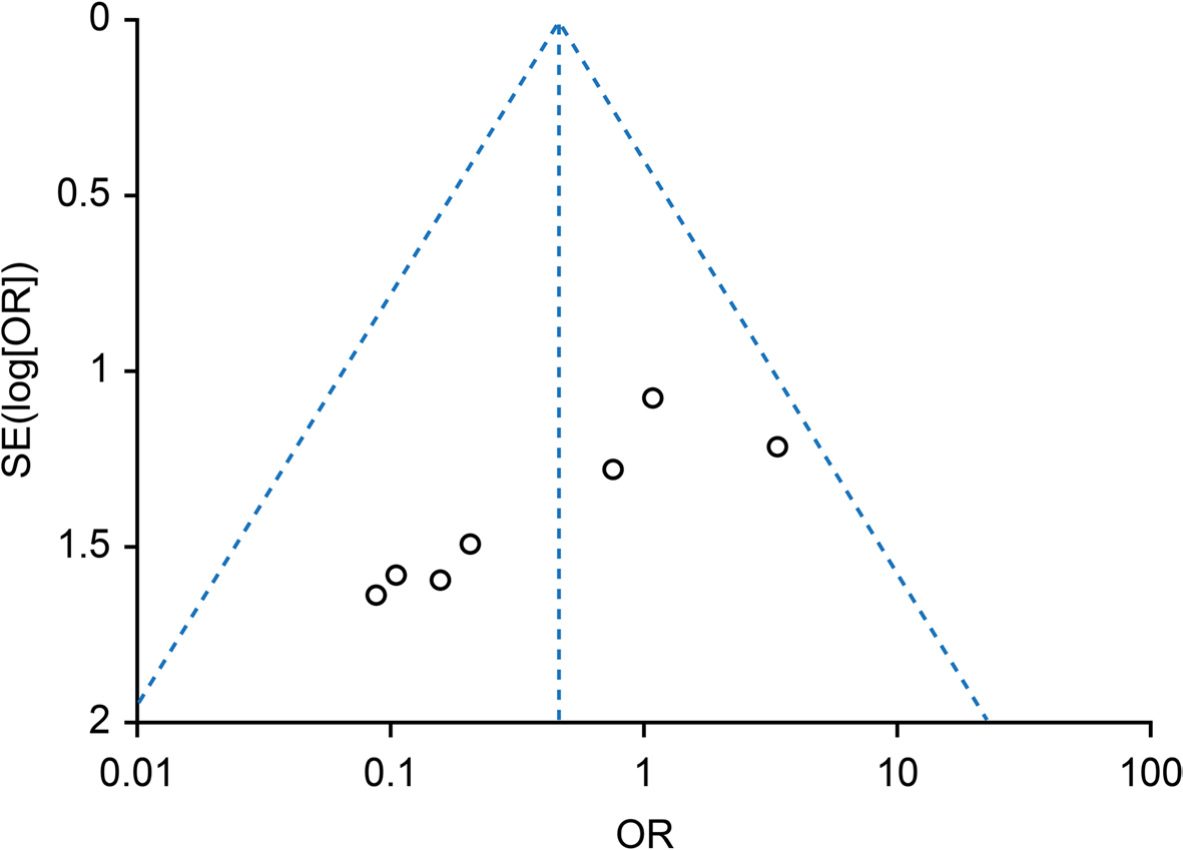
Funnel plot showing the proportion of MHs formed in the FSIP group compared with that in the standard ILM peeling group

The funnel plot in Fig. 7 shows that the proportion of MH formation in MTM was symmetrical. No statistically significant evidence of publication bias was found according to Begg's test (*p* = 0.38) or Egger's test (*p* = 0.47).

### Research gap: absence of comparative studies to date

The only study comparing inverted ILM insertion and the ILM flap with the ABC technique for MTM was not included in this meta-analysis. Our meta-analysis focused on all the novel surgical techniques used for MTM, but no additional comparative studies were found to date. According to Hu et al*.* [25], the MH closure rate was 96% when the ILM flap was used with the ABC technique and 92% when the inverted ILM insertion technique was used (Table 3). This study supports the use of an ILM flap with the ABC technique. However, these findings should be validated in future studies.

**Table 3:** Inverted ILM flap insertion versus the ILM flap insertion with the autologous blood technique

Study design	Study conducted by	No of eyes	Average age in years	Macular Hole (MH) mean diameter	Treatment	Superficial foveal avascular zone (FAZ)	Superficial parafoveal vessel density	Preoperative BCVA (LogMAR)	Postoperative BCVA (LogMAR)	Macular Hole (MH) closure rate	Follow Up months
Retrospective	Hu et al. [25]	25	62.2 ± 11.0	700 μm	ILM flap with Autologous blood technique	0.29 ± 0.08 mm^2^	51.41 ± 2.79%	1.31 ± 0.61	0.68 ± 0.40	96%	3 months
		27	64.4 ± 9.3	700 μm	Inverted ILM insertion	0.73 ± 0.15 mm^2^	43.77 ± 2.71%	1.34 ± 0.39	1.29 ± 0.62	92.5%	3 months

## Discussion

We conducted a meta-analysis of nine studies to evaluate the safety and efficacy of FSIP versus standard ILMP for MTM. A total of 350 eyes were included; 141 eyes were subjected to FSIP treatment, while 209 eyes were treated with standard ILMP. Both groups exhibited similar anatomical outcomes; however, these results support the use of the FSIP technique, which yields better postoperative visual outcomes based on the mean change in logMAR BCVA from baseline. There was a significant difference in the incidence of postoperative MHs between the two groups. Specifically, the postoperative MH rate was lower in the FSIP group (4.25%) than that in the standard ILMP group (8.6%).

According to a meta-analysis by Wu et al. [37], the FSIP group achieved a better postoperative BCVA; however, a comparison of postoperative BCVA was insufficient because differences may have existed at baseline, affecting the postoperative findings. Therefore, we determined the mean change in logMAR BCVA from baseline and observed that the FSIP group had a greater mean improvement than the standard ILMP group. The greater percentage of postoperative MHs in the standard ILMP group might be the reason for the improvement in BCVA in the FSIP group because the visual prognosis is poor in patients with high myopia and MHs [38]. This statement is supported by (Ho et al., 2014) [26], who observed a greater rate of inner/outer segment (IS/OS) line restoration in the FSIP group (9/12) than in the standard ILMP group (1/7), which might have contributed to the better final BCVA. According to Rubinstein et al*.* [39], FTMH occurs after surgery because of ILM-induced trauma. Moreover, they proposed that bleeding occurs in a small area during peeling, leading to postoperative FTMH. In addition, Hussain and Mitra [40] reported that ILM detachment may cause mechanical damage to the inner retina, which may lead to apoptosis of glial cells, resulting in degeneration of neurons and contributing to the formation of postoperative FTMHs. Based on our findings and those of previous research, the unique characteristics of highly myopic eyes, such as increased axial length and structural changes, may have contributed to the greater incidence of FTMH in the ILMP group. These factors may influence the healing process differently in symptomatic eyes compared to healthy eyes.

Importantly, different surgeons have different remnant foveolar ILM diameters in the FSIP. In a study by Wang et al. (2012) [13], the foveal ILM diameter was preserved along the vertical extent of the optic disc. Subsequently, (Elwan et al*.*, 2019) [32] and (Iwasaki et al*.*, 2020) [33] followed the same technique as Shimada et al.; however, the exact diameter was not specified by Elwan et al., while Iwasaki’s group left a 0.5 to 1.0 disc diameter. The size of the preserved ILM was one papillary diameter (PD) in the study by Wang et al*.* (2019) [34], while in the study by Xin et al*.* (2022) [31], an ILM of approximately 1 to 1.5 papillary diameter centered on the fovea centralis was retained, whereas in the standard ILMP group, it was completely removed from 16 eyes. The VA and foveolar structure did not deteriorate over the course of follow-up, which was expected given the assumption that any persisting ILM would function as a preretinal membrane. However, (Ho et al., 2014) [26] and (Tian et al*.*, 2018) [27] chose to reserve ILMs with diameters of 300–500 μm and 500 μm, respectively, because they found that these sizes were suitable for complete foveolar covering and adequate traction release. To the best of our knowledge, the preservation of a smaller section produces better results because the probability of recurrence is lower; however, additional expert surgeries are needed. However, the aforementioned research findings were satisfactory, and it is unlikely that size has an impact on the outcome. Currently, there is no consensus on the extent to which the foveolar ILM should be preserved; thus, further comparative studies and long-term follow-up are needed.

The results of this meta-analysis should be evaluated with caution due to several limitations. One major limitation is that we did not find any comparative studies on novel surgical approaches. After an in-depth search, we found only one comparative study that was not included in the meta-analysis because no other comparative studies were conducted for the same intervention. Future studies should be conducted on these interventions to assess their safety and efficacy. Among the nine included studies, eight were retrospective, and the only prospective study was not randomized. Prospective RCTs with larger sample sizes are required to obtain more conclusive results. Nonetheless, nonrandomized clinical trials contain many uncontrollable factors and lack sufficient data to draw precise conclusions. Moreover, the number of eyes, tamponades, dyes used to stain the ILM, and follow-up times were different in all studies; however, these aspects were not highlighted in this meta-analysis. The follow-up periods of the studies were different and covered an extensive range, from 6 months (Elwan et al*.*, 2019) [32] to more than four years (Ho et al., 2014) [26], which had an impact on the results. In some investigations, the exact time at which the postoperative BCVA was measured was not adequately described; this may have coincided with the end of the follow-up. This meta-analysis included only 350 eyes and aggregated the results from previously published studies; however, these results did not fulfill the standards for an effective meta-analysis. Therefore, additional clinical RCTs with larger sample sizes are warranted. Different FSIP techniques resulted in different sizes of preserved ILMs after surgery, which may have influenced our results. Although these limitations affect the reliability of the conclusions, the results are valuable for demonstrating the superiority of the FSIP technique.

## Conclusion

In conclusion, the present meta-analysis of published studies showed that FSIP is an efficient and safe procedure for the treatment of the initial stages of MTM, with a higher rate of macular reattachment and a lower rate of MH formation than the standard ILMP method. Therefore, FSIP may be the preferred treatment for high myopia conditions such as MTM; however, for patients in the late and advanced stages, we need to implement other novel surgical options, which cannot yet be determined owing to a lack of available studies.

### Supplementary Information

**Supplementary Material 1.**

## Data Availability

The datasets used in this study are included in the main manuscript. Photographs and figures from this study may be released via a written application to the Photographic Laboratory and Clinical Archives Department of the Retina Specialists Unit at Oftalmologia Integral ABC, Medical and Surgical Assistance Institution (nonprofit Organization), Av. Paseo de las Palmas 735 suite 303, Lomas de Chapultepec, Mexico City 11,000, Mexico, and the corresponding author upon request. All analysis files and figures (tiff) can be found in the supplementary file. docx.

## Abbreviations

ABC: Autologous blood cells
AL: Axial length
AMG: Amnios membrane grafting
ART: Autologous retinal transplantation
BCVA: Best-corrected visual acuity
CENTRAL: Cochrane Central Register of Controlled Trials
CFT: Central foveal thickness
CI: Confidence interval
CR: Complete reattachment
ERMs: Epiretinal membranes
FRD: Foveal retinal detachment
FSIP: Fovea-saving internal limiting membrane
FTMH: Full-thickness macular hole
ILMP: Internal limiting membrane peeling
logMAR: Logarithm of the minimum angle of resolution
MB: Macular buckling
MH: Macular hole
MHRD: Macular hole retinal detachment
ML-IILM: Multilayered inverted internal limiting membrane
MINORS: Methodological Index for Nonrandomized Studies
MTM: Myopic traction maculopathy
NOS: Newcastle‒Ottawa Scale
OCT: Optical coherence tomography
ODDs: Odds ratios
PRISMA: Preferred Reporting Items for Systematic Reviews and Meta-analysis
PPV: Pars plana vitrectomy
PM: Pathological myopia
PS: Posterior staphyloma
RCTs: Randomized controlled trials
RD: Retinal detachment
RS: Retinoschisis
SMD: Standardized mean deviation
WMD: Weighted mean difference
