# Supplementary material for: Novel surgical approaches for treating myopic traction maculopathy: a meta-analysis
Source: BMC Ophthalmol. 2024 Mar 5;24:105. doi: 10.1186/s12886-024-03374-0 (PMC10913604; doi:10.1186/s12886-024-03374-0)
Supplement: Supplementary file 1 — Supplementary Material 1. [file 12886_2024_3374_MOESM1_ESM.docx]

**Supplementary file. docx: Myopic Traction Maculopathy Search strategy**

**PubMed,**

(((near sight*) OR (nearsighted) OR (myopia) OR (shortsighted) OR (short sight) OR (myope) OR (myopic defocus))) AND (((macular retinoschisis) OR (lamellar macular hole) or (MRS) or (LMH) or (foveoschisis*) OR (myopic traction maculopathy))) AND (((internal limiting membrane peeling or ILMP) OR (Autologous neurosensorial retina grafting technique*) OR ( Amnios grafting technique*) OR (Lens capsule transplantation*) OR (Multi-layered inverted ILM techniques*) OR (Classical ILM peeling technique*) OR (Fovea-sparing ILM techniques*) OR (Lens capsule transplantation) OR (Multi-layered inverted ILM techniques)))

1 OR 2 OR 3

1 OR 3

1 AND 3

2 AND 3

(Autologous neurosensorial retina grafting technique*) OR ( Amnios grafting technique*) AND (((macular retinoschisis) OR (lamellar macular hole) or (MRS) or (LMH) or (foveoschisis*) OR (myopic traction maculopathy)))

(Lens capsule transplantation*) OR (Multi-layered inverted ILM techniques*) AND (((macular retinoschisis) OR (lamellar macular hole) or (MRS) or (LMH) or (foveoschisis*) OR (myopic traction maculopathy)))

(Standard ILM peeling technique*) OR (Fovea-sparing ILM techniques*) AND (((macular retinoschisis) OR (lamellar macular hole) or (MRS) or (LMH) or (foveoschisis*) OR (myopic traction maculopathy)))

(Lens capsule transplantation) OR (Multi-layered inverted ILM techniques))) AND (((macular retinoschisis) OR (lamellar macular hole) or (MRS) or (LMH) or (foveoschisis*) OR (myopic traction maculopathy)))

**Science Direct**

(Autologous neurosensorial retina grafting technique*) OR ( Amnios grafting technique*) AND (((macular retinoschisis) OR (lamellar macular hole) or (MRS) or (LMH) or (foveoschisis*) OR (myopic traction maculopathy)))

(Lens capsule transplantation*) OR (Multi-layered inverted ILM techniques*) AND (((macular retinoschisis) OR (lamellar macular hole) or (MRS) or (LMH) or (foveoschisis*) OR (myopic traction maculopathy)))

(Standard ILM peeling technique*) OR (Fovea-sparing ILM techniques*) AND (((macular retinoschisis) OR (lamellar macular hole) or (MRS) or (LMH) or (foveoschisis*) OR (myopic traction maculopathy)))

(Lens capsule transplantation) OR (Multi-layered inverted ILM techniques))) AND (((macular retinoschisis) OR (lamellar macular hole) or (MRS) or (LMH) or (foveoschisis*) OR (myopic traction maculopathy)))

**Google Scholar:**

(((near sight*) OR (nearsighted) OR (myopia) OR (shortsighted) OR (short sight) OR (myope) OR (myopic defocus))) AND (((macular retinoschisis) OR (lamellar macular hole) or (MRS) or (LMH) or (foveoschisis*) OR (myopic traction maculopathy))) AND (((internal limiting membrane peeling or ILMP) OR (Autologous neurosensorial retina grafting technique*) OR ( Amnios grafting technique*) OR (Lens capsule transplantation*) OR (Multi-layered inverted ILM techniques*) OR (Classical ILM peeling technique*) OR (Fovea-sparing ILM techniques*) OR (Lens capsule transplantation) OR (Multi-layered inverted ILM techniques)))

(Autologous neurosensorial retina grafting technique*) AND (((macular retinoschisis) OR (lamellar macular hole) or (MRS) or (LMH) or (foveoschisis*) OR (myopic traction maculopathy)))

( Amnios grafting technique*) AND (((macular retinoschisis) OR (lamellar macular hole) or (MRS) or (LMH) or (foveoschisis*) OR (myopic traction maculopathy)))

(Lens capsule transplantation*) AND (((macular retinoschisis) OR (lamellar macular hole) or (MRS) or (LMH) or (foveoschisis*) OR (myopic traction maculopathy)))

(Multi-layered inverted ILM techniques*) AND (((macular retinoschisis) OR (lamellar macular hole) or (MRS) or (LMH) or (foveoschisis*) OR (myopic traction maculopathy)))

(Fovea-sparing ILM techniques*) AND (((macular retinoschisis) OR (lamellar macular hole) or (MRS) or (LMH) or (foveoschisis*) OR (myopic traction maculopathy)))

(Standard ILM peeling technique*) AND (((macular retinoschisis) OR (lamellar macular hole) or (MRS) or (LMH) or (foveoschisis*) OR (myopic traction maculopathy)))

(Lens capsule transplantation) AND (((macular retinoschisis) OR (lamellar macular hole) or (MRS) or (LMH) or (foveoschisis*) OR (myopic traction maculopathy)))

(Multi-layered inverted ILM techniques))) AND (((macular retinoschisis) OR (lamellar macular hole) or (MRS) or (LMH) or (foveoschisis*) OR (myopic traction maculopathy)))

**Keywords search strategy and surgical techniques on Myopic Traction Maculopathy**

| **Medline** | | |
| --- | --- | --- |
| **S.No** | **Keywords** | **Results** |
| **1.** | (macular retinoschisis OR lamellar macular hole OR MRS OR LMH OR foveoschisis* OR myopic traction maculopathy OR MTM) |  |
| **2.** | (internal limiting membrane peeling OR ILMP OR Classical ILM peeling technique* OR Fovea-sparing ILM techniques) |  |
| **3.** | 1 AND 2 |  |
| **4.** | Autologous neurosensorial retina grafting technique* OR Amnios grafting technique*) |  |
| **5.** | 1 AND 4 |  |
| **6.** | (Lens capsule transplantation* OR Multi-layered inverted ILM techniques*) |  |
| **7.** | 1 AND 6 |  |
| **8.** | **3 OR 5 OR 7** |  |
